# Distribution of Hemoglobinopathy Disorders in Al-Kharj Province Based on Data from the Premarital Screening and Genetic Counseling Program

**DOI:** 10.3390/medicina61081458

**Published:** 2025-08-14

**Authors:** Noura Al-Dayan

**Affiliations:** Medical Laboratory, Applied Medical Sciences, Prince Sattam Bin Abdulaziz University, Al-Kharj 16278, Saudi Arabia; n.aldayan@psau.edu.sa

**Keywords:** premarital screening and genetic counseling program, sickle cell disease, α-thalassemia, β-thalassemia, premarital screening

## Abstract

*Background and Objectives*: Hemoglobinopathies are genetic disorders of hemoglobin and are among the most common inherited diseases. The prevalence rates of sickle cell disease and thalassemia in Saudi Arabia are higher than those in other countries in the Middle East. Saudi Arabia has launched many prevention programs such as a premarital screening program, genetic counseling programs, and neonatal screening in order to reduce the incidence of genetic diseases. The former program includes the most common genetic diseases: sickle cell disease and thalassemia. Many studies conducted since the premarital program started have reported a decrease in the prevalence of sickle cell disease and thalassemia. However, all studies focus on large cities, including their subdivisions, but there is a lack of studies on subdivisions specifically. *Materials and Methods*: The aim of this study was to assess the prevalence, 5-year time trend, and distribution of β-thalassemia and sickle cell traits in Al-Kharj province using the data of the PMSGC program during the period from January 2017 to February 2021. *Results*: A total of 21,150 individuals were screened, and 508 were diagnosed with sickle cell disease and thalassemia. Also, we showed that thalassemia was more prevalent than sickle cell disease (66% and 34%, respectively), and there was an increase in β-thalassemia and α-thalassemia. *Conclusions*: Riyadh city’s prevalence rate of β-thalassemia was reported as 7 per 1000, while the current study found a prevalence rate of 5.6 per 1000 in Al-Kharj, which suggests a possible increase as a result of population growth in Al-Kharj province as part of Riyadh city. This study recommends further improvement in preventive measures in high-risk regions, as well as enhanced community awareness, to provide the highest rate of reduction for disorders.

## 1. Introduction

Hemoglobinopathies are genetic disorders in the synthesis of hemoglobin and are among the most common inherited diseases [1]. Global estimates indicate that approximately 300,000–400,000 infants are diagnosed yearly with hemoglobin disorders [2,3]. Thalassemia and sickle cell disease (SCD) are autosomal recessive hemoglobinopathy disorders that have received significant attention from the global public health community because of their impact on increasing mortality and morbidity among affected individuals [4,5]. Thalassemia is further broken down into thalassemia major and thalassemia intermedia. These types of thalassemia require intensive care, resulting in a major disease burden. In general, individuals become clinically asymptomatic in the case of the β-thalassemia (β-thal) trait, which is also known as thalassemia minor [1].

In Saudi Arabia, the prevalence of consanguineous marriages is high, contributing to the propagation of hemoglobinopathies and further leading to ongoing health challenges in the Kingdom. Consanguineous marriages in Saudi Arabia are susceptible to genetic disorders that may be inherited by their offspring [2]. Saudi Arabia has the highest prevalence rates of β-thalassemia and SCD compared to other Middle Eastern countries (0.05–4.50%) [6]. The general statistics organization reported that Saudi Arabia has a high prevalence of thalassemia and sickle cell disease, with an estimated prevalence of 1.5–17% for β-thalassemia and SCD, respectively [3,4]. Preventive screening programs have been adopted in many countries, including Middle Eastern countries, to reduce the prevalence of β-thalassemia and SCD, such as the premarital screening program and genetic counseling (PMSGC), neonatal screening, and pre-implantation genetic diagnosis [7]. In 2001, Saudi Arabia started a program known as the premarital screening program (PMS) to lower the burden of hemoglobinopathy disorders and infectious diseases. The hemoglobinopathy included β-thalassemia and SCD, and infectious diseases included human immunodeficiency (HIV) and hepatitis B and C. However, this program became mandatory in 2004 for both Saudi and non-Saudi couples prior to marriage [5,6,7,8].

National studies conducted in Saudi Arabia have shown a minor decline in the prevalence of SCD, but there are inconsistent reports on the prevalence of β-thalassemia [1,8]. Studies have demonstrated that the highest incidence was in the eastern region (19.5%), followed by Qunfudah (15.8%), Jazan (7.8%), Al-Hasa (3.1%), and Riyadh (0.15%) [9]. Surprisingly, α-thalassemia was not included in all centers of PMS, as the most commonly reported was α-3.7, which is not considered a risky mutation [10]. However, α-thalassemia has been reported to be highly prevalent in the northern and eastern regions, ranging from 0.4% to 5.9%, respectively, and it is co-inherited with sickle cell disease and β-thalassemia [11,12]. Moreover, a 2022 study reported that the α-thalassemia trait had the highest frequency in Makkah city (6.3%) during premarital screening [13]. Alhuthali et al. (2023) [14] identified seven α-thalassemia mutations that might have a large effect on increased mortality and morbidity.

In general, researchers have identified a marked decrease in the number of risky marriages and have predicted a considerable reduction in the genetic disease burden in Saudi Arabia in recent years as a result of premarital genetic counseling (PMSGC) [8]. The PMSGC program has been one of the strongest tools for reducing genetic diseases by identifying risk factors, providing vaccinations, and offering genetic consulting. Although Riyadh city was reported to have the lowest prevalence rates of β-thalassemia, α-thalassemia, and SCD, there is a population increase in Riyadh city which will increase the prevalence rate [9,10].

Al-Saeed et al. [7] performed a study in Saudi Arabia on hemoglobinopathy disorders and included a total of 13 cities in the Kingdom; however, Al-Kharj province was not covered separately. According to the Saudi census of 2022, Al-Kharj has a population of 373,177, which is the largest province population in the capital city; however, the prevalence of hemoglobinopathy disorders has not yet been recorded for Al-Kharj province. This study aims to evaluate the prevalence of hemoglobinopathy in Al-Kharj province over five years and identify the distribution of the disease among males and females. The findings of this study could shed light on the increased incidence, highlighting the need for further improvement in disease prevention efforts and health promotion. In addition, it could help evaluate the effectiveness of genetic counseling programs in achieving a massive reduction in disease burden.

## 2. Materials and Methods

### 2.1. Sample and Population

The current study is a retrospective study that included all samples received at PMS government centers located in Al-Kharj. The samples were collected from individuals, aged 16–55 years, for hemoglobinopathy, HIV, and hepatitis B and C testing. Computerized data were obtained from centers through a digital platform network utilized by health authorities, including the Ministry of Health (MOH), for disease surveillance, reporting, and data collection. The present study only used data uploaded through the network by centers over a five-year period, from January 2017 to February 2021. A total of 21,150 samples were accepted by government centers based on the clinical capacity for hemoglobinopathy and infectious disease testing included in PMS.

### 2.2. Premarital Screening Program and Genetic Counseling Program (PMSGC)

The PMSGC program is supported by an extensive network spanning 13 administrative districts and 131 Ministry of Health Centers throughout Saudi Arabia. PMSGS targets couples pursuing marriage certificates as a prerequisite for screening. The PMS staff gather demographic information, draw blood samples containing the ethylenediaminetetraacetic acid [EDTA] anticoagulant, and offer educational materials that explain the program. Blood samples were used for complete blood count (CBC) (DxH 600, Beckman Coulter, Brea, CA, USA), peripheral blood film analyses, reticulocyte counts, high-performance liquid chromatography (HPLC) (Bio-Rad Variant II, Hercules, CA, USA), hemoglobin electrophoresis (Interlab, Roma, Italy), sickling tests using sodium dithionite and serum ferritin, and virus tests for hepatitis B, HIV, and hepatitis C.

The hemoglobinopathy test results sort individuals from potential couples into healthy, carriers, and affected cases with regard to the genetic make-up of sickle cell disease and/or thalassemia. A safe marriage was declared when both partners or at least one of them was healthy. An at-risk marriage was declared when both partners were carriers or cases (one was a case and the other was a carrier) of sickle cell disease and/or thalassemia. Couples with safe marriage test results were issued instant compatibility certificates, whereas at-risk couples were asked to attend personal genetic counseling meetings before issuing incompatibility certificates. Further, molecular genetic testing was performed by sending the samples to the MOH reference laboratory, which uploads the results to the center pool.

### 2.3. Hemoglobin Electrophoresis Analysis

Hemoglobin electrophoresis samples were examined to detect aberrant hemoglobin bands using capillary electrophoresis, HPLC, or a combination of both. Diagnosis of the β-thalassemia trait is indicated when an individual exhibits a Mean Corpuscular Volume (MCV) of <80 fL and Mean Corpuscular Hemoglobin (MCH) of <27 pg, alongside a hemoglobin A2 level over 3.2%. HPLC tests were conducted regardless of the normal CBC results. Diagnosis of α-thalassemia is indicated when the MCV is <80 fL or hypothermia, the MCH is <26 pg, or both, in the absence of iron deficiency anemia. The couple’s results were interpreted based on standard laboratory diagnostic protocols that were approved by the MOH specifically for the program [15].

### 2.4. Statistical Analysis

Statistical analysis of the PMSGC data was performed using PRISM 10.4.2 and Microsoft Excel 2007, including the analysis of prevalence trends and prevalence rates. Statistical tests included descriptive data as mean and SD for both males and females. In addition, statistical analysis was performed to calculate the percentage of sickle cell anemia and thalassemia.

## 3. Results

### 3.1. Details of Hemoglobinopathy Disorders

The overall study population consisted of 21,150 individuals who attended the PMSCG centers in Al-Kharj province from January 2017 to February 2021. The mean age of the 508 participants was 30.0 + 12.0. Among the 21,150 participants, hemoglobinopathy disorders were confirmed in 2.4% of cases (*n* = 508), with 1.57% (*n* = 333) diagnosed as thalassemia and 0.82% (*n* = 157) as SCD. Also, in terms of gender distribution, 49% were males and 51% were females, with no statistically significant difference between males and females (*p* = 0.01). Table 1 shows the gender distribution of hemoglobinopathy disorders. It is clear that females were predominant in thalassemia (51.1%) and in SCD (51%); in contrast, males were the minority in both conditions, with 48.1% and 49.1%, respectively.

#### 3.1.1. Thalassemia Analysis

Among the total participants, 1.57% were confirmed as having thalassemia traits, of whom 49% were male and 51% were female. Among males, 22.7% of the individuals were under 18 years of age, while 20.75% were aged between 18 and 25 years. The demographics of individuals aged 26 to 35 constituted 18.4%, while those aged 36 to 45 represented 19.63%. The demographics of individuals aged 46–55 years were confirmed to represent 17.06% of the total population. The distribution of females was as follows: 21.18% were under 18 years, 20% were between 18 and 25 years, and 21.76% fell within the 26 to 35 years age range. The percentages of women aged 36–45 and 46–55 were determined to be 20% and 17.06%, respectively. In this study, among the selected individuals of both genders, 21.9% were under 18 years of age, 20.1% fell within the 18–25 and 26–35 age range, 19.8% were aged 36–45, and 18% were within the 46–55 age range. The majority of thalassemia traits were identified in individuals under 18 years of age, encompassing both men and women, as well as a mixed population. In females, thalassemia was notably higher in the age group of 26–35 years at 21.7%, compared to 21.2% in those under 18 years. Table 2 provides the percentages and prevalence of the thalassemia traits observed across different age groups. A two-way ANOVA showed a significant association (*p* < 0.0001) between the male and female groups. Further analysis was conducted to examine thalassemia prevalence.

#### 3.1.2. Screening of Thalassemia

**Figure 1:**
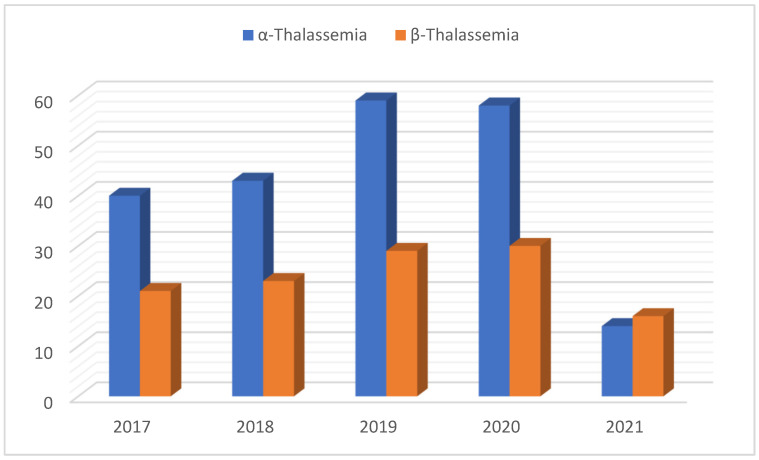
Trends of α- and β-thalassemia traits from 2017 to 2021.

This study diagnosed 333 patients with thalassemia, further dividing them into α- and β-thalassemia groups. α-thalassemia accounted for 64.3% of cases, while β-thalassemia represented 35.7%. This study was conducted over a period of five years, i.e., from January 2017 to February 2021. Table 3 shows the prevalence of both α- and β-thalassemia over a period of five years along with a graph representation, which is displayed in Figure 1. The prevalence of α-thalassemia was recorded to be 18.7%, 20.1%, 27.6%, 27.1%, and 6.5%, respectively, between 2017 and 2021, while β-thalassemia prevalence was found to be 17.6%, 19.3%, 24.4%, 25.2%, and 13.4%, respectively. The highest recorded prevalence of α- and β-thalassemia was documented in 2019 and 2020, respectively.

### 3.2. Prevalence Rate of SCD

In this study, 175 patients were diagnosed with SCD over a duration of five years in Al-Kharj province. The proportion of males and females with SCD was 49.1% and 50.9%, respectively. The analysis was conducted based on age-wise criteria for both males and females. Among men, 9.7% of the participants were younger than 18 years old, 10.3% were 18 to 25 years old, 13.1% were 26 to 35 years old, 8.6% were 36 to 45 years old, and 7.4% were 46 to 55 years old. Among women, 11.4% were younger than 18 years old, 14.9% were 18 to 25 years old, 7.4% were 26 to 35 years old, 10.3% were 36 to 45 years old, and 6.9% were 46 to 55 years old. The highest prevalence of SCD was observed in males aged 26–35 years (13.1%) and in females aged 18–25 years (14.9%). The details are listed in Table 4.

### 3.3. Prevalence of SCD Between 2017 and 2021

Table 5 confirms the documented prevalence of SCD between the years 2017 and 2021 in Al-Kharj city. The prevalence of SCD was found to be 18.9%, 20%, 21.7%, 25.7%, and 13.7%, respectively, in the years 2017 to 2021. However, the highest prevalence of SCD was 21.7% in 2019.

### 3.4. Prevalence Rate in Hemoglobinopathy Disorders

Overall, the prevalence rate estimated for α-thalassemia in the study population over the 5-year period was 10 per 1000, and the prevalence rate for β-thalassemia was 5.6 per 1000. In addition, the prevalence rate of SCD was estimated to be 8 per 1000 among the 21,150 people examined. Details are shown in Table 6. Among the 21,150 subjects, 23.6% of the participants were recruited between 2017 and 2020, but only 5.4% were enrolled in 2021. The prevalence of thalassemia (α and β) was 12.2%, 13.2%, 17.6%, and 26%, and in SCDs, it was 6.6%, 7%, 7.6%, 9%, and 20%, respectively.

## 4. Discussion

In Saudi Arabia, hemoglobinopathies are considered the most prevalent disorders, although their prevalence varies among cities in the Kingdom. Variations in thalassemia and SCD rates can be attributed to multiple factors, including genetic and environmental factors, along with cultural aspects and diseases in the families. Hemoglobin disorders are common monogenic disorders worldwide, in which α-thalassemia, β-thalassemia, SCD, and hemoglobin-E diseases are considered to be more prevalent. All genetic disorders associated with hemoglobin are referred to as hemoglobinopathies and recognized as single-gene disorders [9]. The rate of consanguineous marriage in Saudi Arabia was documented to be high at approximately 56%, which indicates that genetic diseases are common [10].

Saudi Arabia initiated the concept of a premarital screening program, also known as the Healthy Marriage program, in 2001, and it was strictly implemented in 2004. The MOH started and planned this program to identify genetic risks (β-thalassemia and SCDs) and reduce the risk of infectious diseases (HIV and hepatitis B and C) among Saudi couples before marriage. Screening for these diseases can help prevent infectious diseases, identify their prevalence in couples before marriage, and promote a healthier life for their offspring. α-thalassemia was not included in most of the PMS [12]. In Saudi Arabia, the most important issue in marriages is consanguinity, which has a powerful impact on marrying within the family. With the help of genetic counselors, Saudi couples benefit from the PMS program’s knowledge and importance. Finally, after the PMS certificate is issued, it allows them to proceed with their marriage [6,11,12].

The PMS program is mainly beneficial for documenting genetic disorders, particularly in Saudi couples with high rates of consanguineous marriages. Autosomal recessive disorders such as thalassemia and SCDs are prevalent in Saudi Arabia. Abnormalities in thalassemia and SCDs will lead to anemia and organ damage, as well as other complications, and there are many risks affecting offspring when both parents are carriers of these mutations, further compounded by the disadvantages of consanguineous unions [13,14].

The prevalence of hemoglobinopathies in Saudi Arabia varies from city to city. The study by Alsaeed et al. [7] established the highest prevalence of β-thalassemia in Jazan (32%), followed by the eastern region (23.7%), Makkah (14.4%), and Al-Baha (13.2%). Furthermore, the distribution of confirmed cases was as follows: 8.1% in Madina, 7.6% in the Northern Border, 7% in Riyadh, 6.7% in Asir, 6.3% in Tabuk, 4% in Qasim, 3.3% in Hail, 2.9% in Al-Jouf, and 2.4% in Najran [7]. An updated study in the southern region has confirmed a prevalence of 7% in hemoglobinopathies [15]. Another study from Jeddah has documented hemoglobinopathy screening results, reporting a 7.2% prevalence of β-thalassemia and 9.4% for SCD [16]. A recent study in Riyadh city has documented an SCD prevalence of 4.7% and a β-thalassemia prevalence of 1.2% [17]. All previous studies in PMS have focused on β-thalassemia and SCD, and only a few mention α-thalassemia [11]. Moustafa et al. [13] have reported the highest frequency of α-thalassemia in Makkah city, with a PR of around 6.3%. Molecular screening and identification of α-thalassemia contribute to prenatal diagnosis and realistic genetic counseling in regions at risk for thalassemia, yet these services are not available at most MOH centers [12].

A significant occurrence of hemoglobinopathies has been recorded in Saudi Arabia among the GCC nations, followed by Oman, which exhibits a notable prevalence of the sickle cell trait. In contrast, the UAE, Qatar, and Bahrain demonstrate a lower frequency of these conditions [17].

The aim of this study was to screen the PMS in Al-Kharj city. Overall, 21,150 individuals participated in this study over a duration of five years, between 2017 and 2021. The study results confirmed that 2.4% of the participants had hemoglobinopathy disorders, in which 1.57% were confirmed for thalassemia and 0.82% for SCDs. The prevalence of hemoglobinopathies was reported to be low in the central region of Saudi Arabia when compared to other regions in the Kingdom. Studies have focused on β-thalassemia and SCDs, with none of the meta-analysis studies recording α-thalassemia because it is considered not risky [18]. A few studies have demonstrated that α-thalassemia mutations are distributed around Saudi Arabia, and some of these are high-risk and might have a large impact on increased mortality and need more attention [14]. A limitation of the current study is the lack of molecular identification for α-thalassemia.

## 5. Conclusions

This study confirms a hemoglobinopathy prevalence of 1.57% in Al-Kharj city. This is the first documented study in the Kingdom, and future studies are recommended to investigate the prevalence and genetic mutations of hemoglobinopathies in detail in Al-Kharj city.

## Figures and Tables

**Table 1:** Gender distribution among individuals diagnosed with hemoglobinopathy disorders.

Gender	Thalassemia Trait	SCD	Hemoglobinopathy Disorders
Males	163 (48.9%)	86 (49.1%)	249 (49%)
Females	170 (51.1%)	89 (50.9%)	259 (51%)

**Table 2:** Age group distribution for males and females diagnosed with Thalassemia traits.

Gender	Total Cases	<18 Years	18–25 Years	26–35 Years	36–45 Years	46–55 Years
Males	163 (100%)	37 (22.70%)	33 (20.75%)	30 (18.40%)	32 (19.63%)	31 (19.02%)
Females	170 (100%)	36 (21.18%)	34 (20%)	37 (21.76%)	34 (20%)	29 (17.06%)

**Table 3:** Positive results confirmed for α- and β-thalassemia traits during the period of 2017–2021.

Year	α-Thalassemia Trait (*n* = 214)	β-Thalassemia Trait (*n* = 119)
2017	40 (18.7%)	21 (17.6%)
2018	43 (20.1%)	23 (19.3%)
2019	59 (27.6%)	29 (24.4%)
2020	58 (27.1%)	30 (25.2%)
2021	14 (6.5%)	16 (13.4%)

**Table 4:** Distribution of age groups among male and female individuals diagnosed with SCD.

Gender	<18 Years	18–25 Years	26–35 Years	36–45 Years	46–55 Years
Males	17 (9.7%)	18 (10.3%)	23 (13.1%)	15 (8.6%)	13 (7.4%)
Females	20 (11.4%)	26 (14.9%)	13 (7.4%)	18 (10.3%)	12 (6.9%)

**Table 5:** Year-wise SCD prevalence in Al-Kharj province from 2017 to 2021.

Year-Wise	Diagnosis of Sickle Cell Traits (*n* = 175)
2017	33 (18.9%)
2018	35 (20.0%)
2019	38 (21.7%)
2020	45 (25.7%)
2021	24 (13.7%)

**Table 6:** Prevalence rates of α-/β-thalassemia and SCD in Al-Kharj province between 2017 and 2021.

	Population Screened	α-Thalassemia	β-Thalassemia	
Year	Total Participants (*n* = 21,150)	Positive Subjects (*n* = 119)	Confirmed Subjects (*n* = 214)	PR
2017	5000 (23.6%)	40 (18.7%)	21 (17.6%)	12.20%
2018	5000 (23.6%)	43 (20.1%)	23 (19.3%)	13.20%
2019	5000 (23.6%)	59 (27.6%)	29 (24.4%)	17.60%
2020	5000 (23.6%)	58 (27.1%)	30 (25.2%)	17.60%
2021	1150 (5.4%)	14 (6.5%)	16 (13.4%)	26%

## Data Availability

The original contributions presented in this study are included in the article. Further inquiries can be directed to the corresponding author.

## Abbreviations

The following abbreviations are used in this manuscript:

PMSGC	Premarital Screening and Genetic Counseling Program
SCD	sickle cell disease
PMS	premarital screening program
HIV	human immunodeficiency
HPLC	high-performance liquid chromatography
MOH	Ministry of Health
CBC	complete blood count
